# Assessment of subjective emotional valence and long-lasting impact of life events: development and psychometrics of the Stralsund Life Event List (SEL)

**DOI:** 10.1186/s12888-018-1649-3

**Published:** 2018-04-18

**Authors:** Johanna König, Andrea Block, Mathias Becker, Kristin Fenske, Johannes Hertel, Sandra Van der Auwera, Kathleen Zymara, Henry Völzke, Harald Jürgen Freyberger, Hans Jörgen Grabe

**Affiliations:** 1 Department of Psychiatry and Psychotherapy, University Medicine Greifswald, Ellernholzstraße 1-2, 17489 Greifswald, Germany; 2 Department of Health Sciences, Institute of Sociology of Health and Physical Activity, University of Potsdam, Potsdam, Germany; 3 Department of Psychiatry and Psychotherapy, Helios Clinic, Stralsund, Germany; 4 German Centre of Neurodegenerative Diseases (DZNE), Site Rostock/Greifswald, Greifswald, Germany; 5 Institute for Community Medicine, University Medicine Greifswald, Greifswald, Germany

**Keywords:** Positive life events, Negative life events, General population, Emotional valence, Depressive disorder

## Abstract

**Background:**

Life events (LEs) are associated with future physical and mental health. They are crucial for understanding the pathways to mental disorders as well as the interactions with biological parameters. However, deeper insight is needed into the complex interplay between the type of LE, its subjective evaluation and accompanying factors such as social support. The “Stralsund Life Event List” (SEL) was developed to facilitate this research.

**Methods:**

The SEL is a standardized interview that assesses the time of occurrence and frequency of 81 LEs, their subjective emotional valence, the perceived social support during the LE experience and the impact of past LEs on present life. Data from 2265 subjects from the general population-based cohort study “Study of Health in Pomerania” (SHIP) were analysed. Based on the mean emotional valence ratings of the whole sample, LEs were categorized as “positive” or “negative”. For verification, the SEL was related to lifetime major depressive disorder (MDD; Munich Composite International Diagnostic Interview), childhood trauma (Childhood Trauma Questionnaire), resilience (Resilience Scale) and subjective health (SF-12 Health Survey).

**Results:**

The report of lifetime MDD was associated with more negative emotional valence ratings of negative LEs (OR = 2.96, *p* < 0.0001). Negative LEs (b = 0.071, *p* < 0.0001, β = 0.25) and more negative emotional valence ratings of positive LEs (b = 3.74, *p* < 0.0001, β = 0.11) were positively associated with childhood trauma. In contrast, more positive emotional valence ratings of positive LEs were associated with higher resilience (b = − 7.05, *p* < 0.0001, β = 0.13), and a lower present impact of past negative LEs was associated with better subjective health (b = 2.79, *p* = 0.001, β = 0.05). The internal consistency of the generated scores varied considerably, but the mean value was acceptable (averaged Cronbach’s alpha > 0.75).

**Conclusions:**

The SEL is a valid instrument that enables the analysis of the number and frequency of LEs, their emotional valence, perceived social support and current impact on life on a global score and on an individual item level. Thus, we can recommend its use in research settings that require the assessment and analysis of the relationship between the occurrence and subjective evaluation of LEs as well as the complex balance between distressing and stabilizing life experiences.

**Electronic supplementary material:**

The online version of this article (10.1186/s12888-018-1649-3) contains supplementary material, which is available to authorized users.

## Background

Negative life events (LEs) have been associated with enhanced biological stress reactions and enhanced risk of physical and mental diseases. For example, as part of gene-environment-interactions (GxE interactions), childhood and adulthood trauma have been described as an important environmental factor [1–7]. Thus, Grabe et al. [7] demonstrated that the s-allele of the 5-HTTLPR polymorphism was highly associated with higher depression scores only in subjects who reported both childhood and adulthood traumatic LEs. On the other hand, positive LEs have been associated with lower biological stress levels and increased resilience [8–12]. Hence, Haeffel and Vargas [9] reported positive LEs to buffer the negative impact of negative LEs on depressive symptoms. In addition, various studies have demonstrated that the impact of LEs on long-term health outcomes depends on the experience’s characteristics, e.g., experience time as well as individual protective resources [11, 13–17]. Thus, Kleiman et al. [18] reported optimistic subjects to be less affected by negative LEs, and Asselmann et al. [19] observed that coping efficacy mediated the impact of negative LEs on mental health. Further, Brown and McGill [16, 17] found subjects with low self-esteem to benefit less from the buffering effect of positive LEs, and Staufenbiel et al. [11] reported positive LEs to moderate the association between social support and hair cortisol levels. However, it is unclear whether the experience, and thus the number of LEs, or the subjective emotional perception of the LE is more important in this regulation.

The substantial importance of LEs for long-term health has stimulated the development of scales measuring the experience of positive and negative LEs. For example, one of the first known scales is Meyer’s *Life Chart*, which uses a lifeline to collect and organize the onset and duration of LEs as well as physical and mental diseases [20, 21]. Likewise, Caspi et al. [22] developed the *Life History Calendar (LHC)*, which records LEs and diseases graphically linked to a calendar. However, both scales focus on the LE and life history but do not include an evaluation of the emotional valence of the LEs [20–22]. The *Social Readjustment Rating Scale (SRRS)* assesses 43 LEs associated with fixed life change units, which correspond to standardized ratings of subjective stress reactions and are summed to evaluate the risk of disease [23]. In contrast, the German language *Munich Event List (MEL)* assesses the subjective stress level and emotional valence for each LE individually [24, 25]. However, both the *SRRS* and the *MEL* focus on the assessment and evaluation of LEs but do not include explicitly an assessment of accompanying dimensions such as social support. Within the *Life Events and Difficulties Schedule (LEDS),* the interviewee is guided to a comprehensive LE story covering the surrounding situation during the LE experience [26, 27]. Later, these stories are rated regarding the “emotional arousal”, “general contextual threat” and “specific aspects of the threat” by an evaluator who was uninvolved during the interview [26–28]. Within the German language *Inventar zur Erfassung lebensverändernder Ereignisse (ILE)*, all LEs are rated by the individual in terms of controllability and predictability as well as personal coping skills and perceived social support [29, 30]. Another German-language scale, the *Leipziger Ereignis- und Belastungsinventar (LEBI)*, focusses on the impact of LEs on major aims in life and assesses social support, subjective burden and subjective controllability [31]. However, the *SRRS*, *LEDS*, *ILE* and *LEBI* only assess the individual burden caused by the LEs but do not assess the subjective emotional valence (positive/negative/neutral) associated with the LEs.

Within the “Study of Health in Pomerania” (SHIP), a comprehensive health assessment, our working group aimed to investigate the influences of LEs and GxE interactions on mental and physical health outcomes. This research approach requires an advanced assessment tool to evaluate various aspects of LEs:

1. We aimed to assess positive, negative and neutral LEs covering the whole lifespan.
2. Similar to the *LHC,* we aimed to assess the time of occurrence, the frequency and the duration of LEs using a life history method to be able to relate the LEs to mental and physical diseases.
3. In contrast to the SRRS, we aimed to assess the subjective burden caused by the LEs individually, as it is unclear to what extent this could be represented properly by a priori standardized values [28]. In addition to what is assessed in the *LEDS*, *ILE* and *LEBI*, we aimed to assess the subjective emotional valence of all LEs (positive/negative/neutral).
4. In addition to what is assessed in the *MEL,* we aimed to assess the perceived social support received during the LE experience, as accompanying circumstances have been demonstrated to influence the impact of LEs on future life and health status [5, 11, 28, 32].

No single measurement was available that covered all the abovementioned requirements. Hence, we set out to develop a new interview that aimed to optimize and extend the already existing measurements: the *Stralsund Life Event List (Stralsunder Lebensereignisliste, SEL)*. The *SEL* is a standardized interview assessing not only the occurrence and frequency of LEs but also the perceived social support and the impact of past LEs on current life. In contrast to existing measurements, the emotional valence of LEs was not determined a priori but was rated individually by the interviewees. These emotional valence ratings were assessed for all LEs at the time of their occurrence as well as at the time of the interview. The present article describes the development, structure, first descriptive statistics and preliminary validity and reliability data of the *SEL*.

## Methods

### Study populations

We analysed data from the study “Life Events and Gene-Environment-Interaction in Depression” (SHIP-LEGEND), which is a sub-sample of the “Study of Health in Pomerania” (SHIP) [33]. SHIP is a two-stage stratified population-based cohort study conducted in West Pomerania that comprises a baseline sample of 4308 Caucasian participants (SHIP-0, 20-79 years), who were drawn from local registers between 1997 and 2001. Three follow-up measurements (SHIP-1: 2002-2006, SHIP-2: 2008-2012, SHIP-3: 2015-2017) were conducted. SHIP-LEGEND was conducted in parallel to SHIP-2. All participants from the baseline sample (SHIP-0) still alive in 2006 were asked to participate in SHIP-LEGEND. A total of 2400 subjects participated in SHIP-LEGEND between 2007 and 2010. SHIP-LEGEND comprised a comprehensive psychological assessment by fully qualified psychologists or advanced psychology students based on diagnostic interviews for mental disorders and LEs (SEL) as well as questionnaires that were sent to the participant’s home before the interviews. The interviews occurred in the Departments of Psychiatry and Psychotherapy in Greifswald and Stralsund or at the participant’s home. The presented data are based on a rectified sample (*n* = 2265) that excluded participants whose response behaviours were rated as highly unreliable or inconsistent by the interviewer (*n* = 106) and/or participants who did not complete the interview (*n* = 117).

All participants had given written informed consent. The study protocol and methods of SHIP and SHIP-LEGEND were approved by the local Institutional Review Board of the University of Greifswald and conformed to the principles of the Declaration of Helsinki.

For preliminary reliability analyses, a second, clinical sample (*n* = 19) was drawn. All participants were patients in the day hospital of the Department of Psychiatry and Psychotherapy of the University Medicine Greifswald in 2012 and 2013. To assess the test-retest reliability, half of the participants (*n* = 9) were re-interviewed approximately 28 days (mean (M) = 28.7, standard deviation (SD) = 4.7) after the initial interview by the same interviewer. The remaining participants (*n* = 10) were re-interviewed approximately two days (M = 1.8, SD = 0.4) after the initial interview by another interviewer. All participants in the reliability sample gave written informed consent. The reliability sample was used to estimate the inter-rater and test-retest reliabilities. The corresponding analyses are presented in the Additional file 1 and Additional file 2: Table S3, Additional file 3: Table S4 and Additional file 4: Table S5.

### Stralsund life event list (Stralsunder Lebensereignisliste, SEL)

The SEL comprises 81 LEs structured in 14 sections (Additional file 5: Table S1). The created LE items are partially based on existing LE measurements [22, 24, 26, 29, 31], supplemented by further positive LEs and sample-specific, major historical LE questions concerning, for example, forced displacement in the post-war era (World War II) and the German reunification in 1990. In addition to 70 standardized LEs, 11 open questions were added, giving the participant the opportunity to report additional previously unmentioned LEs.

For each LE, the participants were asked to report if they had ever experienced the given LE (*occurrence of LE*). If a LE had not been experienced, the interviewer continued with the next one. If a LE had been experienced, the frequency and respective onsets of the LE were coded. To standardize the onset coding process, onsets were categorized in 5-year periods starting with 5 to 10 years up to 81 to 85 years. If there were multiple experiences of the same LE, only the one with the highest subjective meaning for the participant was rated in detail. The exact age at the occurrence was noted (Additional file 6: Figure S1). Further, to improve the coding of the time of the occurrence (*occurrence time coding*), LEs were embedded in the context of developmental milestones (e.g., graduation, marriage, childbirth). For each experienced LE, participants rated the *emotional valence* of the LE at the time of the occurrence and at the time of the interview on a 5-point scale (1 = very positive, 2 = positive, 3 = neutral, 4 = negative, 5 = very negative). Similar to the *emotional valence* ratings, the perceived *social support* at the time of the occurrence was rated (0 = no social support desired, 1 = no social support, 2 = minimal social support, 3 = moderate social support, 4 = considerable social support). The rating scales for *emotional valence* and perceived *social support* were given to the participants during the interview. Finally, the participants were asked to decide whether the past LE still had an impact on their emotional and social life currently (*present impact*). If a LE was not applicable (e.g., items about children if the participant had never had children), the item was skipped. For a coding example, see Additional file 6: Figure S1. Note that there are LEs where some of the ratings were not appropriate.

Because this article aims to provide a first overview of the SEL and its psychometric criteria, all analyses will focus on lifespan measurements. Further, to ensure the comparability of the SEL scores, open questions (unstandardized items) were excluded from all analyses.

### Further interviews and psychometric data

To associate LEs with pathogenic mechanisms of mental and physical diseases, valid diagnoses are essential. Therefore, prior to the SEL interview, the Munich Composite International Diagnostic Interview [34] was used. The M-CIDI is a structured and standardized interview evaluating the individual lifetime prevalence of mental diseases according to the internationally used classification systems ICD-10 and DSM-IV. The M-CIDI has been shown to be highly reliable and valid in assessing the onset and duration of the disorders [35]. To assess subjective health, the SF-12 Health Survey [36] was used. Additionally, depressive symptoms and childhood trauma were assessed using the Beck Depression Inventory-II [37] and Childhood Trauma Questionnaire [38], respectively. Resilience and social support were assessed by the Resilience Scale [39] and the Social Support Questionnaire [40, 41], respectively. All questionnaires have been shown to be highly reliable [36, 41–44].

### Statistical analyses

For descriptive analyses, metric variables are reported in means (M) and standard deviations (SD). Categorical variables are reported in frequencies. All *p*-values (*p*) reported are two-tailed. Statistical analyses were performed in STATA/SE 14.

#### Classification process of LEs

To aggregate LE information but sustain inter-individual and inter-LE comparability, we a posteriori applied a classification process that resulted in three classes of LEs (valence categories) typifying positive, negative and neutral LEs. The classification process was based on the average *emotional valence* rating for each LE within the SHIP-LEGEND sample. Every LE’s mean *emotional valence* at the time of its occurrence rated retrospectively was tested for the deviation from 3 (3 = neutral) using a one-sample t-test. Means that were significantly smaller were classified as positive, and means that were significantly higher, as negative. Means that did not vary significantly from 3 were classified as neutral. As the *emotional valence* at the time of the occurrence was not assessed in seven items where this information was not appropriate (items S9, B15, K28, L35, A47, F50 and D57), these LEs were typified as positive, negative and neutral using the mean *emotional valence* rating at the time of the interview. Only four LEs (H2, K22, A43, G63) were classified as neutral. Thus, the neutral valence category was neglected in further analyses.

#### SEL scores

Since we aimed to aggregate the rating information assessed by the SEL, we calculated scores based on the classification process described. For every participant, the *basic number of LEs* was calculated for positive and negative LEs. The *basic number of LEs* represents the number of different LEs experienced over the whole lifespan. Further, the *proportion* of positive and negative LEs in fact rated as positive or negative, respectively, was calculated to determine the individual *emotional valence* at the time of the occurrence. To develop a score that reveals not only the number of different experienced LEs (*basic number of LEs*) but also the total number of experienced LEs including repeated occurrences of the same LE, a *total frequency of LEs* was calculated per valence category for every participant by adding up the individual frequency of each experienced LE. To compare the *emotional valence* ratings at the time of the interview inter-individually, the mean individual *emotional valence* for positive and negative LEs was determined for every participant. Therefore, a quotient of the sum of the individual *emotional valence* ratings of experienced LEs and the individual *basic number of LEs* was calculated for each valence category. This procedure was repeated for the *social support* and *present impact* ratings.

#### Verification analyses

To verify the explanatory power of the SEL, well-studied relations between LEs, childhood trauma, health and individual resources were replicated. Using univariate multivariable linear regression analyses, the associations between the *basic number, total number, proportion, emotional valence* at the time of the interview and *present impact* of positive and negative LEs were considered with the following questionnaires: SF-12 Health Survey (SF-12) [36], Resilience Scale (RS-25) [39], and Childhood Trauma Questionnaire (CTQ) [38]. To validate the *social support* scores, the Social Support Questionnaire (F-SozU) [40] was used. Using a univariate multivariable linear logistic regression, the impact of the *basic number, total number, proportion, emotional valence* and *present impact* of LEs on the probability of reporting a lifetime diagnosis of major depressive disorder (MDD) was also assessed. As depressive symptoms repeatedly have been associated with recognition biases concerning LE reports [45–48], the BDI-II total score was used as a covariate for all analyses [37] in addition to age, sex and educational level. Hence, 12 multivariable regression analyses were calculated for each verification scale.

#### Internal consistency

To analyse the internal consistency of the SEL scores, Cronbach’s alpha was calculated using the SHIP-LEGEND sample [49, 50]. As sufficient observations are needed for calculation, nine LEs reported by less than 5% of the sample (*n* = 113) were excluded from analysis.

Statistical analyses concerning the inter-rater and test-retest reliabilities are presented in the Additional file 1.

## Results

### Descriptive statistics

The following analyses are based on 2265 participants (47.7% male, M = 55.3 years, SD = 13.9 years, age range: 29-89 years). A sample description is provided in Table 1. The BDI-II, CTQ, RS-25, SF-12 and F-SozU scores are similar to scores reported for the general population [41–43, 51, 52]. The descriptive statistics of all SEL items including the mean *frequencies*, mean *emotional valence* ratings, mean *social support* ratings and *present impact* ratings are presented in Additional file 7: Table S2. The descriptive statistics of the SEL scores are provided in Table 2.

**Table 1:** Descriptive statistics for 2265 analysed participants of SHIP-LEGEND

	SHIP-LEGEND		No Lifetime MDD^a^		Lifetime MDD^a^		*p*-value
	Missing (#)		Missing (#)		Missing (#)		
N	–	2265	–	1876	–	386	–
Male (%)	0	47.7	0	51.2	0	30.8	< 0.0001^h^
Age (years)	0	55.3 (13.9)	0	55.8 (14.1)	0	52.6 (12.8)	< 0.0001^g^
Education (%)	3		2		1		0.011^h^
< 10 years		26.9		28.1		21.0	
10 years		53.1		52.8		54.2	
> 10 years		20.0		19.0		24.6	
Lifetime MDD (%)^a^	3	17.0	–	–	–	–	–
Current Depressive Sympoms (BDI-II)^b^	102	6.3 (7.1)	82	5.3 (5.8)	20	11.3 (9.9)	< 0.0001^g^
Childhood Trauma (CTQ)^c^	101	43.9 (11.2)	83	43.0 (5.8)	18	48.3 (15.1)	< 0.0001^g^
Resilience (RS-25)^d^	44	145.5 (18.4)	35	147.2 (17.2)	9	137.5 (21.6)	< 0.0001^g^
Subjective Health (SF-12)^e^	161	49.3 (7.1)	130	50.1 (6.6)	31	45.6 (8.3)	< 0.0001^g^
Social Support (F-SOzU)^f^	43	4.3 (0.6)	34	4.3 (0.6)	9	4.1 (0.7)	< 0.0001^g^

^a^*MDD* Major Depressive Disorder, ^b^*BDI-II* Beck Depression Inventory-II, ^c^*CTQ* Childhood Trauma Questionnaire

^d^*RS-25* Resilience Scale, ^e^*SF-12* SF-12 Health Survey, ^f^*F-SozU* Social Support Questionnaire, ^g^p-value of Welch t-test

^h^*p*-value of Fisher’s exact test

**Table 2:** Descriptive statistics of the SEL scores for 2265 analysed participants of SHIP-LEGEND

	Positive			Negative		
	*N*	M	(SD)	*N*	M	(SD)
Basic number of Life Events	2265	14.75	(2.21)	2265	9.02	(3.88)
Proportion of Life Events rated as positive/negative^a^	2265	0.83	(0.12)	2262	0.77	(0.19)
Total Number of Life Events	2265	13.73	(4.41)	2265	14.10	(8.31)
Emotional Valence (Time of the Interview)^b^	2264	1.88	(0.32)	2260	3.65	(0.52)
Present Impact^c^	2264	0.79	(0.16)	2260	0.30	(0.25)
Social Support (Undesired)^d^	386	17.04%		139	6.14%	
Desired^e^	1873	2.11	(1.13)	2118	2.45	(1.02)

^a^Rating not available for all life event items, ^b^Rating: 1 (very positive) – 5 (very negative), ^c^Rating: 0 (no) vs. 1 (yes)

^d^Rating: 0 (no social support desired), ^e^participants reporting undesired social support excluded; Rating: 1 (no social support) – 4 (much social support)

### Verification analyses

**Fig. 1:**
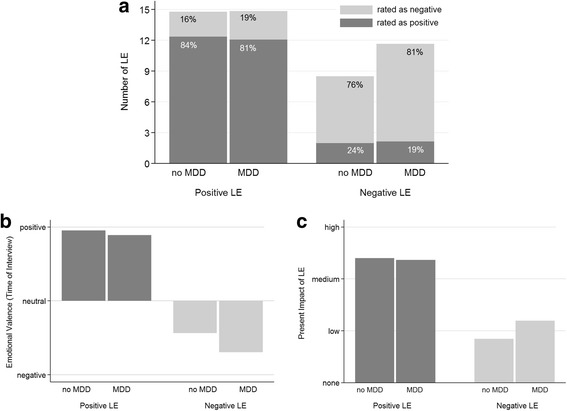
Associations of lifetime major depressive disorder (MDD) and life event (LE) Ratings. All LE ratings are stratified for lifetime major depressive disorder (MDD) as well as for positive and negative LEs according to the classification process. **a** Proportions of LEs actually rated as positive and negative, respectively. *Participants with lifetime MDD have equal proportions of positively rated positive LEs (OR = 0.63, 95%-CI = 0.22;1.75, p = 0.317) but higher proportions of negatively rated negative life events (OR = 4.61, 95%-CI = 2.20;9.64, p < 0.001).* **b** Emotional valence ratings at the time of the interview*. For participants reporting lifetime MDD ratings are more negative for negative LEs (OR = 2.96, 95%-CI = 2.29;3.84, p < 0.001) but do not differ from participants without lifetime MDD for positive LEs (OR = 1.26, 95%-CI = 0.85;1.85, p = 0.252).* **c** The present impact of past LEs. *Participants with lifetime MDD are reporting more impact of past negative LEs on their current emotional and social life (OR = 2.80, 95%-CI = 1.69;4.63, p < 0.001) but do not differ from participants without lifetime MDD for positive LEs (OR = 0.68, 95%-CI = 0.31;1.50, p = 0.343)*

Participants reporting lifetime MDD reported higher *basic numbers of negative LEs* (odds ratio (OR) = 1.17, 95%-CI = 1.13;1.21, *p* < 0.001) than those reported by participants without lifetime MDD. Lifetime MDD was also associated with more negatively rated life events in both valence categories, even though only the proportion of negatively rated negative LEs reached significance (OR = 4.61, 95%-CI = 2.20;9.64, *p* < 0.001); see Fig. 1a. Further, lifetime MDD was associated with a more negative current emotional valence rating of negative LEs (OR = 2.96, 95%-CI = 2.29;3.84, *p* < 0.001) and a higher present impact of negative LEs (OR = 2.80, 95%-CI = 1.67;4.63, *p* < 0.001) (Fig. 1b and c). Similarly, childhood trauma was associated with more negative LEs (b = 0.71, 95%-CI = 0.59;0.83, *p* < 0.001, β = 0.25) and reduced proportions of positively rated positive LEs (b = − 7.12, 95%-CI = − 10.97;-3.27, *p* = 0.0003, β = − 0.08). However, childhood trauma was related to neither the basic number of positive LEs (b = 0.08, 95%-CI = − 0.15;0.31, *p* = 0.487, β = 0.02) nor to the proportion of negatively rated negative LEs (b = 0.74, 95%-CI = − 1.62;3.09, *p* = 0.540, β = 0.01). Further, with increasing severity of childhood traumas, positive LEs were currently rated more negatively (b = 3.74, 95%-CI = 2.28;5.19, *p* < 0.001, β = 0.11), but this was not true for negative LEs (b = 0.50, 95%-CI = − 0.37;1.38, *p* = 0.259, β = 0.02). Interestingly, childhood trauma was negatively associated with the perceived social support during the LE occurrence for positive (b = − 1.34, 95%-CI = − 1.81;-0.86, *p* < 0.001, β = 0.13) and negative LEs (b = − 2.15, 95%-CI = − 2.62;-1.68, *p* < 0.001, β = 0.20). In contrast to lifetime MDD and childhood trauma, high resilience was associated with higher proportions of positively rated positive LEs (b = 11.62, 95%-CI = 5.91;17.33, *p* < 0.001, β = 0.08) as well as increased present impact of positive life events (b = 9.93, 95%-CI = 5.61;14.25, *p* < 0.001, β = 0.09) but was not related to any score for negative LEs. Higher proportions of positively rated positive LEs (b = 2.79, 95%-CI = 1.07;4.51, *p* = 0.001, β = 0.05) and fewer reported negative LEs (b = − 0.12, 95%-CI = − 0.18;-0.07, *p* < 0.001, β = 0.07) were associated with better subjective health. Accordingly, better subjective health was associated with a lower present impact of negative LEs (b = − 2.14, 95%-CI = − 2.99;-1.29, *p* < 0.001, β = − 0.07). Higher social support reported in the F-SozU questionnaire was associated with higher SEL *social support* scores for both positive (b = 0.05, 95%-CI = 0.03;0.08, *p* < 0.001, β = 0.10) and negative LEs (b = 0.12, 95%-CI = 0.09;0.14, *p* < 0.001, β = 0.20). For an overview of the regression coefficients, see Table 3.

**Table 3:** Regression coefficients of the verification analyses

	MDD^a^	CTQ^b^		RS-25^c^		SF-12^d^		F-SozU^e^	
	OR(95%-CI)	b(95%-CI)	β	b(95%-CI)	β	b(95%-CI)	β	b(95%-CI)	β
Positive Life Events									
Basic number of Life Events	1.14(1.06;1.22)***	0.08(−0.15;0.31)	0.02	0.06(−0.28;0.41)	0.01	0.17(0.07;0.28)***	0.05	0.04(0.03;0.05)****	0.15
Proportion of Life Events rated positive	0.63(0.22;1.75)	−7.12(−10.97;-3.27)***	−0.08	11.62(5.91;17.33)****	0.08	2.79(1.07;4.51)**	0.05	0.70(0.50;0.90)****	0.14
Total Number of Life Events	1.07(1.04;1.10)****	0.28(0.17;0.39)****	0.11	0.13(−0.03;0.29)	0.03	−0.03(− 0.08;0.02)	−0.02	0.01(0.01;0.02)****	0.08
Emotional Valence (Time of the Interview)^f^	1.26(0.85;1.85)	3.74(2.28;5.19)****	0.11	−7.05(−9.20;-4.90)****	−0.13	−0.95(−1.61;-0.30)**	0.04	− 0.52(− 0.59;-0.45)****	−0.28
Present Impact^g^	0.68(0.31;1.50)	−3.94(−5.96;-0.11)*	−0.04	9.93(5.6;14.025)****	0.09	1.22(−0.08;2.52)	0.03	0.46(0.31;0.61)****	0.12
Social Support^h^	1.00(0.89;1.13)	−1.34(−1.81;-0.86)****	−0.13	−0.08(− 0.76;0.60)	0.00	−0.02(− 0.22;0.19)	0.00	0.05(0.03;0.08)****	0.10
Negative Life Events									
Basic number of Life Events	1.17(1.13;1.21)****	0.71(0.59;0.83)****	0.25	−0.06(−0.25;0.12)	− 0.01	−0.12(− 0.18;− 0.07)****	-0.07	−0.01(− 0.01;-0.00)*	−0.05
Proportion of Life Events rated negative	4.61(2.20;9.64)****	0.74(−1.62;3.09)	0.01	−1.44(−4.94;2.05)	−0.02	0.21(−0.84;1.26)	0.01	0.06(−0.06;0.18)	0.02
Total Number of Life Events	1.04(1.03;1.06)****	0.30(0.24;0.35)****	0.22	−0.04(− 0.13;0.04)	−0.02	− 0.06(− 0.08;-0.03)****	−0.07	0.00(− 0.01;0.0)	−0.04
Emotional Valence (Time of the Interview)^f^	2.96(2.29;3.84)****	0.50(−0.37;1.38)	0.02	−0.69(−1.99;0.60)	− 0.02	−0.46(− 0.85;-0.07)*	−0.03	0.03(− 0.02;0.07)	0.03
Present Impact^g^	2.80(1.69;4.63)****	0.89(−1.037;2.82)	0.02	2.65(−0.20;5.51)	0.04	−2.14(−2.99;-1.29)****	−0.07	0.03(− 0.07;0.13)	0.01
Social Support^h^	1.02(0.89;1.16)	−2.15(− 2.62;-1.68)****	−0.20	0.03(− 0.67;0.74)	0.00	− 0.02(− 0.23;0.19)	0.00	0.12(0.09;0.14)****	0.20

All analyses adjusted for age, sex, educational level and BDI-II. * *p*-value < 0.05; ** *p*-value < 0.01; *** *p*-value < 0.001; **** *p* < 0.0001

^a^Lifetime Major Depressive Disorder (yes vs. no), ^b^Childhood Trauma Questionnaire, ^c^Resilience Scale, ^d^SF-12 Health Survey, ^e^Social Support Questionnaire

^f^Rating: 1 (very positive) – 5 (very negative), ^g^Rating: 0 (no) vs. 1 (yes)

^h^Participants reporting undesired social support excluded; Rating: 1 (no social support) – 4 (much social support)

### Reliability

#### Internal consistency

The SHIP-LEGEND sample was used to assess Cronbach’s alpha (α). The calculated α varied between 0.38 and 0.94 for positive and negative scores (Table 4). Only the *emotional valence* of positive LEs (α = 0.73), the present impact of positive LEs (α = 0.69) and both total numbers of LE frequency (α = 0.38 resp. α = 0.54) had an α lower than 0.76, which is the average α over all SEL scores for our data.

**Table 4:** Cronbach’s alpha^a^ of the SEL scores

	Positive	Negative
Basic number of Life Events	0.78	0.91
Total Number of Life Events	0.38	0.54
Emotional Valence (Time of the Interview)	0.73	0.87
Present Impact	0.69	0.88
Social Support	0.83	0.94
Averaged	0.68	0.83

^a^SHIP-LEGEND sample (*N* = 2265) without Life Events reported by less than 5% of the sample (*n* = 113)

The results of the inter-rater and test-retest analyses are presented in the Additional file 1 and in Additional file 2: Tables S3, Additional file 3: Table S4 and Additional file 4: Table S5.

## Discussion

The SEL is a standardized interview that assesses positive and negative LEs over the whole lifespan using a life history method [22]. Thereby, the SEL combines an assessment of the time of the LE *occurrence* and the LE *frequency* with an assessment of the subjective *emotional valence*. In contrast to existing LE scales, within the SEL, the *emotional valence* is not determined a priori by normative assumptions but rather individually rated by each participant. To aggregate LE information for practical reasons, an empirical classification process was performed a posteriori to identify positive, negative and neutral LEs based on the mean *emotional valence* ratings of our large, population-based sample (*N* = 2265). Further, the desired and perceived *social support* as well as the *present impact* of past LEs on current life were assessed.

### Verification analyses

For verification analyses, we assessed well-established relations between LEs and mental health. As suggested by former research, lifetime MDD was associated with more negative LEs [48]. However, we demonstrated that not only the kind of LE (positive/negative) but also the subjective evaluation of the LE impacts future health outcomes. Thus, MDD was more strongly associated with the *emotional valence* ratings of LEs than with the *occurrence of LEs* per se [48, 53, 54]. In line with former research describing hyperactive emotional responses in maltreated subjects [55–57], we observed more negative *emotional valence* ratings also to be associated with more childhood trauma. In contrast, higher scores on the RS-25 and SF-12, assessing resilience and subjective health, respectively, were associated with more positive *emotional valence* ratings, which is supported by former research regarding LEs, chronic strain and resilience [58–60].

### Internal consistency

Tavakol and Dennick [50] summarized that alphas between 0.70 and 0.90 are desirable. According to these authors, lower values might be based on a low number of items, low inter-item correlations or a heterogeneous construct [50]. The latter two factors are desired and inherent facts of the SEL scores, as the SEL was designed to cover a high variety of LEs, and the classification process aimed to aggregate but not unitise the SEL items. Nevertheless, for the SHIP-LEGEND sample, Cronbach’s alpha was smaller than 0.70 only for the *total number of positive and negative LEs*. This might be a result of the coding process used for repeated LE occurrences. Because rare LEs are better remembered than typical ones [61], LE frequencies higher than 10 were universally coded as 11. This led to a ceiling effect and an inaccurate estimate of the *total number of LEs* that probably reduced Cronbach’s alpha values. In future research, this rating might be reconstructed for future assessments, which might also enhance the reliability of these scores.

### Limitations

First, the whole interview took about one to two h depending on the interviewee’s life history, willingness for disclosure and cognitive speed. Especially for subjects with low concentration and endurance levels, this led to a decrease in quality with ongoing interview time. Further, recall biases had to be considered, as autobiographical memory is reconstructive and affected by cognitive mechanisms [62, 63]. We intended to improve the recall accuracy using a life history method [22]. Second, the classification procedure was based on the entire population-based sample (*N* = 2265) including participants reporting lifetime MDD and high current depressive symptoms. Research suggests that a cognitive vulnerability for depression leads to a more negative interpretation of LEs in depressed individuals [45–47]. However, only 17% (*n* = 386) of the SHIP-LEGEND sample reported lifetime MDD, and the descriptive statistics of the whole sample were more similar to participants not reporting lifetime MDD. To compensate for a cognitive response bias due to current depressed mood, we adjusted the analyses for current depressive symptoms. Nevertheless, the classification procedure is highly dependent on the study sample. Thus, one must mention that the SHIP-LEGEND sample is a subsample of the SHIP-0, which has the risk for selection bias (e.g., mortality, willingness for participation) and an elevated age range. Third, seven items (S9, B15, K28, L35, A47, F50 and D57) indicated a positive *emotional valence* through the wording of the description of the LE, as reported by 70-98% of the SHIP-LEGEND participants. This indicated both low specificity and low sensitivity, and future assessments should decide whether to reformulate or exclude these items. Fourth, in addition to several striking effect sizes (e.g., *proportion* of negatively rated negative LEs and lifetime MDD, *basic number* of negative LEs and childhood trauma, *emotional valence rating* of positive LEs at the time of the interview and resilience), we also observed small effect sizes, which are significant due to the large study sample size. Odds ratios and beta values determine the effect sizes depending on the used covariates within the regression model. All verification analyses were adjusted for the BDI-II total score, as depression has been associated with a recognition and evaluation bias [45–48]. As the scales used to verify the SEL are related to depression as well as, current depressive symptoms [7, 59, 60, 64–68], the inclusion of the BDI-II total score in the analyses may have diminished the effect sizes. Further, a putative selection bias might have impacted the results. As we analysed the second follow-up of a general population sample, healthier subjects might be relatively overrepresented. Hence, the variance within the responses on the verification scales would be decreased, which would also have an impact on the effect sizes. Fifth, the SEL must still be compared to other LE measurements. As several LE items used in the SEL are shared with other instruments [22, 24, 26, 29, 31], we generally assume a good agreement between the SEL and other life event scales. However, validation is still required. Finally, all analyses conducted are cross-sectional. Thus, causality should be discussed with caution. Further, the value of the SEL for prospective analysis remains to be elucidated. Longitudinal analyses are conceivable as soon as the data from the third follow-up measurement of the SHIP studies (SHIP-3) are available. Further, note that no adjustments for multiple tests were used in the present analyses. Due to the large sample size of the SHIP-LEGEND sample, the *p*-values of the verification analyses were very small and robust against multiple testing.

### Future implications

Within a therapeutic setting, the SEL could be used to assess LEs occurring immediately before a disease onset. Although we focussed on the whole lifespan in the present manuscript, the SEL could be adapted to assess any period of life of interest. Within the SHIP-LEGEND study, the SEL interview was used to assess the 12-month and 5-year periods before the interview and the 5-year period before the onset of the first subclinical depressive episode as well as the first and the worst clinical depressive episodes. Furthermore, the 5-year period before the onset of a obsessive-compulsive disorder and the 5-year period before and after an initial panic attack were assessed. By integrating the subjective *emotional valence* and *present impact*, the most afflicting LEs could be selected as a central theme within the therapeutic process. Moreover, in LE research, the SEL could be a valuable measurement to associate subjective emotional ratings with future health status, to identify subgroups of LEs as well as their differential impacts on diseases, and to integrate personal circumstances and LE evaluations. Within our own working group, the SEL will be used for longitudinal analyses regarding physical and mental health based on future follow-up measurements of the SHIP studies. Moreover, the SEL can inform research on the interplay between life experiences and biological factors, e.g., in GxE interactions (e.g., [7, 64, 69, 70]).

## Conclusion

Previous studies have demonstrated the impact of subjective emotional evaluations in addition to objective valence assessments [71], and LEs have been repeatedly demonstrated to enhance the risk of various mental and physical diseases, especially if interacting with genetic dispositions [4, 8, 10, 13, 14]. The SEL not only enables the testing of the impact of LEs on the pathology of diseases but also integrates the impact of the experience with the individual emotional valence and subjective long-lasting life impact. Thus, the SEL could help generate and test new models of the pathogenesis of various disorders and facilitate research on potential mechanisms of stress balance. Moreover, the SEL is adaptable not only in the lifetime period measured but also in the individuality of the appraisal. The described SEL scores are only one way of analysing the data. More individualized analyses can be generated and are desirable in future research. Therefore, we recommend the SEL as a valuable instrument in life event research on disorder and health outcomes.

## Additional files

Additional file 1: Reliability Analyses. To estimate the inter-rater and test-retest reliability, a small second, clinical sample was used. The methods, results, discussion and limitations of these analyses are presented within this supplementary material. (PDF 283 kb)

Additional file 2: **Table S3.** Inter-rater and test-retest intraclass coefficient (ICC) reliabilities of the SEL scores. * *p*-value < 0.05; ** *p*-value < 0.01; *** *p*-value < 0.001. ^b^Averaged Intraclass Coefficient based on the reliability samples (*N* = 10 resp. 9). (PDF 183 kb)

Additional file 3: **Table S4.** Occurrence agreement of the life event items. ^a^Based on the inter-rater reliability sample (*N* = 10), ^b^Based on the test-retest reliability sample (*N* = 9), ^c^Number of subjects with identical occurrence ratings (yes/no), ^d^Sum of life events with identical occurrence ratings for all subjects, ^e^Sum of life events with identical occurrence ratings for 90% of the subjects (*n* = 9 resp. *n* = 8). (PDF 261 kb)

Additional file 4: **Table S5.** Reliability of the occurrence time coding of selected life events^1^. ^1^ Life events were selected if present at both time points of reliability measurement for at least 5 interviewees. * *p*-value < 0.05; ** *p*-value < 0.01; *** *p*-value < 0.001 (PDF 199 kb)

Additional file 5: **Table S1.** Life event items and corresponding abbreviations of the SEL interview. ^a^ open questions. (PDF 398 kb)

Additional file 6: **Figure S1.** Coding Example. A fictive person moved out of its childhood home twice (18 and 28 years). The most important experience was at the age of 18. The person had one subclinical depressive episode at the age of 32 and an initial panic attack at the age of 20. Hence, life event ratings concern the age of 18 for the whole lifespan and the 5-year period before the initial panic attack. For the 5-year period before the subclinical depressive episode, life event ratings concern the age of 28. LT = Lifetime; 12 = 12 months prior to the interview; 5Y = 5 years prior to the interview; 1SuD = 5 years prior to the first subclinical depressive episode; 1DE = 5 years prior to a clinical depressive episode; WDE = 5 years prior to the worst depressive episode; OCD = 5 years prior to an obsessive-compulsive disorder; IPA- = 5 years prior to an initial panic attack; IPA+ = 5 years after an initial panic attack. (TIF 249 kb)

Additional file 7: **Table S2.** Descriptive statistics of the life event items for 2265 analysed participants of SHIP-LEGEND. Note: Due to the life event items some information is unavailable. Due to a low using frequency and in desire to improve the inter-individual comparability, open questions were not considered in the classification process. ^a^without missing values, ^b^open questions, ^c^Rating: 1 (very positive) – 5 (very negative), ^d^Rating: 0 (no social support desired), 1(no social support) – 4 (much social support). (XLSX 25 kb)

## Abbreviations

BDI-II: Beck Depression Inventory II
CI: Confidence Interval
CTQ: Childhood Trauma Questionnaire
F-SozU: Social Support Questionnaire
GxE interaction: Gene-Environment Interaction
ICC: Intraclass Coefficient
LE: Life Event
M: Mean
MDD: Major Depressive Disorder
p: *p*-value
RS-25: Resilience Scale
SD: Standard Deviation
SEL: Stralsund Life Event List
SF-12: SF-12 Health Survey
SHIP: Study of Health in Pomerania
SHIP-LEGEND: Study of Health in Pomerania - Life Events and Gene-Environment-Interaction in Depression
α: Cronbach’s Alpha
κ: Cronbach’s Kappa
