# Supplementary material for: Assessment of subjective emotional valence and long-lasting impact of life events: development and psychometrics of the Stralsund Life Event List (SEL)
Source: BMC Psychiatry. 2018 Apr 18;18:105. doi: 10.1186/s12888-018-1649-3 (PMC5907180; doi:10.1186/s12888-018-1649-3)
Supplement: Supplementary file 1 — Reliability Analyses. To estimate the inter-rater and test-retest reliability, a small second, clinical sample was used. The methods, results, discussion and limitations of these analyses are presented within this supplementary material. (PDF 283 kb) [file 12888_2018_1649_MOESM1_ESM.pdf]

Assessment of Subjective Emotional Valence and Long-Lasting Impact of Life Events: Development and Psychometrics of the Stralsund Life Event List (SEL)

Johanna König, Andrea Block, PhD, Mathias Becker, Kristin Fenske, Johannes Hertel, Sandra Van der Auwera, PhD, Kathleen Zymara, Henry Völzke, MD, Harald Jürgen Freyberger, MD, Hans Jörgen Grabe, MD

SUPPLEMENTARY MATERIAL A - Reliability Analyses

METHODS

Statistical Analyses

For inter-rater and test-retest analyses, the SEL interviews of the reliability sample (N=19) were used. For both analyses, an agreement of the *occurrence* of each LE was calculated. To assess the accuracy of the *occurrence time coding*, several LEs were selected. To achieve a suitable statistical power, the selection was based on the criteria that a given LE had to be present at both time points of the reliability measurement in at least half of the interviewees (n=5). These selection criteria resulted in 23 LEs for the inter-rater reliability assessment and 16 LEs for the test-retest reliability assessment (supplementary table S4). The accuracy of the *occurrence time coding* was calculated by assessing the agreement of the 5-year period coded using weighted Cohen's kappa values [1–3]. To assess the inter-rater and test-retest reliability of the SEL scores, averaged intraclass correlations (ICC) were calculated based on answer agreements between the averages of ratings from several raters [1, 4, 5].

RESULTS

Inter-Rater Reliability. Ten participants (20.0% male; age: M=40.9, SD=12.2) from the reliability sample were re-interviewed by a second interviewer two days (M=1.8, SD=0.4) after the initial interview. On average, the *occurrence* agreement between the two interviews was 94.3%, with 42 LEs (60%) being reported as identical in both interviews (table S3). Except for four items (H6, B16, L31 and T69), the weighted Cohen's kappa (κ) for *occurrence time coding* was higher than 0.65 (Table S4), which indicates a substantial to almost perfect accuracy [1, 2]. As depicted in table 4, except for the *present impact* of past positive LEs (ICC=0.41, p=0.208), all inter-rater ICCs

for the SEL scores were higher than 0.69 and thus can be described as good; all scores for negative LEs were higher than 0.75 and were, thus, excellent [1]. For more detailed information, see tables 4, S3 and S4.

Test-Retest Reliability. Nine participants (55.6% male; M=45.2, SD=13.3) from the reliability sample were re-interviewed by the same interviewer 28 days (M=28.7, SD=4.7) after the initial interview. On average, the *occurrence* agreement between the two interviews was 94.3%, with 28 LEs (40%) being reported as identical in both interviews (table S3). The mean κ for the *occurrence time coding* was 0.68 (Table S4). Only the items H6 (κ=0.36, p=0.097) and W52 (κ=0.24, p=0.232) had a κ lower than 0.4, which was defined as moderate [1]. Except for the *present impact* of past negative LEs (ICC=0.29, p=0.308), all ICCs of the SEL scores (0.73-0.97) indicated excellent test-retest reliabilities [1]. For more detailed information, see tables 4, S3 and S4.

DISCUSSION

Inter-Rater and Test-Retest Reliability. The lowest inter-rater agreement (60%) was observed for item U78 "serious accident, terrible experiences or catastrophes as listed". Reporting traumatic experiences might be emotionally demanding. Thus, the responses to item U78 might depend not only on the pure facts of the occurrence but also on the relationship with and faith in the interviewer. Accordingly, the test-retest agreement of item U78 was much higher (89%) than the inter-rater agreement. Low inter-rater reliabilities were also observed for item H6 "move out of childhood home" and L31 "complete traineeship/pass examinations". Both items had long latency periods between the LE occurrence and the interview, which has been demonstrated to reduce recall accuracy [6]. Cohen's kappa for item W52 was critically low only in the test-retest analysis, which indicates a high test-retest-interval sensitivity. Overall, inter-rater reliability, which features the comparability and equality of the ratings of different interviewers, was high and thus indicated high implementation and evaluation objectivity. The test-retest reliability was comparable to the inter-rater reliability for the SEL scores. Please note that the test-retest and inter-rater reliability analyses were based on a small clinical sample. Hence, these preliminary results need to be interpreted with caution and require future research using larger sample sizes.

Limitations. The preliminary test-retest and inter-rater reliability analyses were based on a small clinical sample with all subjects suffering from mental disorders and receiving psychiatric or psychotherapeutic treatments at the time of the interviews. As these interventions might specifically have changed the emotional evaluations of past LEs and their impact on present wellbeing between the interviews, we would expect even better reliability in healthy subjects. However, it is highly important to test this hypothesis in future research. Moreover, according to Brown and Harris (1982) [6], a “fall-off” regarding the number of reported LEs is expected with increasing time between interviews, particularly for LEs that are low in threat and salience. Nevertheless, our preliminary reliability indices were good but need to be supported by further reliability studies in healthy subjects with larger sample sizes.

SUPPLEMENTARY REFERENCES

1. Hallgren KA. Computing Inter-Rater Reliability for Observational Data: An Overview and Tutorial. Tutor Quant Methods Psychol. 2012;8:23–34.
2. Landis JR, Koch GG. The measurement of observer agreement for categorical data. Biometrics. 1977:159–74.
3. Cohen J. Weighted kappa: Nominal scale agreement provision for scaled disagreement or partial credit. Psychol Bull. 1968;70:213–20.
4. Rousson V, Gasser T, Seifert B. Assessing intrarater, interrater and test-retest reliability of continuous measurements. Stat Med. 2002;21:3431–46. doi:10.1002/sim.1253.
5. Stata Corp. Stata 12 Base Reference Manual. College Station, Texas: Stata Press; 2011.
6. Brown GW, Harris TO. Fall-off in the reporting of life events. Soc Psychiatry. 1982;17:23–8. doi:10.1007/BF00583889.
