# Supplementary material for: Assessment of subjective emotional valence and long-lasting impact of life events: development and psychometrics of the Stralsund Life Event List (SEL)
Source: BMC Psychiatry. 2018 Apr 18;18:105. doi: 10.1186/s12888-018-1649-3 (PMC5907180; doi:10.1186/s12888-018-1649-3)
Supplement: Supplementary file 2 — Table S3. Inter-rater and test-retest intraclass coefficient (ICC) reliabilities of the SEL scores. * p-value < 0.05; ** p-value < 0.01; *** p-value < 0.001. bAveraged Intraclass Coefficient based on the reliability samples (N = 10 resp. 9). (PDF 183 kb) [file 12888_2018_1649_MOESM2_ESM.pdf]

Table S3: Inter-rater and test-retest intraclass coefficient (ICC) reliabilities of the SEL scores

	**Positive**		**Negative**	
	Inter-Rater ICC[b]	Test-Retest ICC[b]	Inter-Rater ICC[b]	Test-Retest ICC[b]
Basic number of Life Events	0.97***	0.97***	0.92***	0.82**
Proportion of Life Events rated as positive/negative	0.73*	0.77*	0.77*	0.73*
Total Number of Life Events	0.94***	0.97***	0.83**	0.76*
Emotional Valence (Time of the Interview)	0.85**	0.92***	0.96***	0.93***
Present Impact	0.41	0.92**	0.75*	0.29
Social Support	0.70*	0.82*	0.94***	0.92**
**Averaged**	0.77	0.89	0.86	0.74

* p-value < 0.05; ** p-value < 0.01; *** p-value <0.001

[b]Averaged Intraclass Coefficient based on the reliability samples (N=10 resp. 9)
