# Supplementary material for: Assessment of subjective emotional valence and long-lasting impact of life events: development and psychometrics of the Stralsund Life Event List (SEL)
Source: BMC Psychiatry. 2018 Apr 18;18:105. doi: 10.1186/s12888-018-1649-3 (PMC5907180; doi:10.1186/s12888-018-1649-3)
Supplement: Supplementary file 3 — Table S4. Occurrence agreement of the life event items. aBased on the inter-rater reliability sample (N = 10), bBased on the test-retest reliability sample (N = 9), cNumber of subjects with identical occurrence ratings (yes/no), dSum of life events with identical occurrence ratings for all subjects, eSum of life events with identical occurrence ratings for 90% of the subjects (n = 9 resp. n = 8). (PDF 261 kb) [file 12888_2018_1649_MOESM3_ESM.pdf]

Table S4: Occurrence agreement of the life event items

	Inter-Rater Reliability[a]		Test-Retest Reliability[b]	
Life Event	N[c]	%	N[c]	%
H1	10	100.0	9	100.0
H2	10	100.0	9	100.0
H3	9	90.0	7	77.8
H4	9	90.0	9	100.0
H5	9	90.0	5	55.6
H6	10	100.0	8	88.9
S8	9	90.0	6	66.7
S9	9	90.0	7	77.8
S10	9	90.0	8	88.9
B12	10	100.0	7	77.8
B13	10	100.0	9	100.0
B14	10	100.0	9	100.0
B15	10	100.0	8	88.9
B16	7	70.0	9	100.0
B17	10	100.0	8	88.9
B18	10	100.0	9	100.0
K20	10	100.0	9	100.0
K21	10	100.0	9	100.0
K22	10	100.0	9	100.0
K23	9	90.0	8	88.9
K24	10	100.0	8	88.9
K25	10	100.0	9	100.0
K26	9	90.0	7	77.8
K27	10	100.0	9	100.0
K28	10	100.0	8	88.9
L30	10	100.0	9	100.0
L31	10	100.0	8	88.9
L32	10	100.0	9	100.0
L33	8	80.0	8	88.9
L34	10	100.0	9	100.0
L35	10	100.0	7	77.8
L36	10	100.0	8	88.9
A38	10	100.0	8	88.9
A39	8	80.0	8	88.9
A40	10	100.0	9	100.0
A41	9	90.0	7	77.8
A42	9	90.0	8	88.9
A43	9	90.0	8	88.9
A44	9	90.0	8	88.9
A45	10	100.0	9	100.0
A46	9	90.0	9	100.0
A47	7	70.0	7	77.8

F49	10	100.0	8	88.9
F50	8	80.0	8	88.9
W52	10	100.0	8	88.9
W53	7	70.0	5	55.6
J55	10	100.0	8	88.9
D57	9	90.0	8	88.9
D58	9	90.0	7	77.8
D59	10	100.0	7	77.8
D60	9	90.0	8	88.9
G62	9	90.0	7	77.8
G63	10	100.0	9	100.0
G64	10	100.0	7	77.8
G65	9	90.0	9	100.0
T67	10	100.0	9	100.0
T68	10	100.0	9	100.0
T69	9	90.0	7	77.8
T70	10	100.0	8	88.9
T71	10	100.0	9	100.0
T72	10	100.0	8	88.9
T73	9	90.0	9	100.0
O74	10	100.0	9	100.0
O75	9	90.0	7	77.8
O76	10	100.0	9	100.0
O77	10	100.0	8	88.9
U78	6	60.0	8	88.9
U79	10	100.0	8	88.9
R80	10	100.0	9	100.0
R81	10	100.0	9	100.0
∑ discrepancy = 0[d]	42	60.0	28	40.0
∑ discrepancy = 1[e]	21	30.0	26	37.1
Ø agreement	9.4	94.3	8.1	90.0

[a]Based on the inter-rater reliability sample (N=10), [b]Based on the test-retest reliability sample (N=9), [c]Number of subjects with identical occurrence ratings (yes/no), [d]Sum of life events with identical occurrence ratings for all subjects, [e]Sum of life events with identical occurrence ratings for 90% of the subjects (n=9 resp. n= 8)
