# Supplementary material for: Assessment of subjective emotional valence and long-lasting impact of life events: development and psychometrics of the Stralsund Life Event List (SEL)
Source: BMC Psychiatry. 2018 Apr 18;18:105. doi: 10.1186/s12888-018-1649-3 (PMC5907180; doi:10.1186/s12888-018-1649-3)
Supplement: Supplementary file 4 — Table S5. Reliability of the occurrence time coding of selected life events1. 1 Life events were selected if present at both time points of reliability measurement for at least 5 interviewees. * p-value < 0.05; ** p-value < 0.01; *** p-value < 0.001 (PDF 199 kb) [file 12888_2018_1649_MOESM4_ESM.pdf]

Table S5: Reliability of the occurrence time coding of selected life events[1]

Abbr.	Life Event<br>Short Description	Inter-Rater Reliability<br>κ(SD)	Test-Retest Reliability<br>κ(SD)
H3	Arguments with parents	0.65(0.30)*	-
H5	Arguments between parents	0.87(0.31)**	-
H6	Move out of childhood home	0.43(0.28)	0.36(0.27)
S8	Broke up of long-term friendship	0.74(0.30)**	
S10	Cut back on leisure activities	-	0.46(0.31)
B12	Heartache	0.83(0.30)**	-
B13	Serious relationships	0.67(0.28)**	0.92(0.33)**
B14	Marriage	1.00(0.32)***	0.78(0.33)**
B16	Serious crisis in relationships	0.42(0.25)*	0.69(0.30)*
B17	Separations and divorces	0.96(0.31)**	0.71(0.31)*
B18	Unfaithful relationship	1.00(0.32)***	-
K21	Birth of healthy children	0.98(0.32)***	0.97(0.33)**
K25	Move out of children	-	0.90(0.33)**
L31	Complete traineeship/Pass examinations	0.35(0.29)	0.57(0.29)**
L36	Inability to start desired course of training	0.95(0.31)**	0.80(0.32)**
A39	Promotion/Professional Success	-	0.48(0.30)
A42	Unemployment/Inability to work	0.95(0.32)**	0.64(0.33)*
A46	Overburdened by professional tasks	0.69(0.32)*	1.00(0.33)**
F49	Financial problems	0.66(0.32)*	-
W52	Moving/Build a house/Renovation	0.67(0.31)*	0.24(0.32)
G62	Serious bodily illness or accident (self)	0.69(0.31)*	-
G64	Serious bodily illness or accident (others)	0.96(0.31)**	0.80(0.33)**
T69	Death of important relatives or friends	0.52(0.32)*	0.55(0.33)*
T70	Death of a pet	0.96(0.31)***	-
O74	Physical/Sexual abuse by a stranger	0.82(0.31)**	-
O77	Witnessed the abuse of a close person	0.91(0.31)**	-
	**Averaged**	0.77(0.20)	0.68(0.22)

[1] Life events were selected if present at both time points of reliability measurement for at least 5 interviewees.

* p-value < 0.05; ** p-value < 0.01; *** p-value < 0.001
