# Supplementary material for: Assessment of subjective emotional valence and long-lasting impact of life events: development and psychometrics of the Stralsund Life Event List (SEL)
Source: BMC Psychiatry. 2018 Apr 18;18:105. doi: 10.1186/s12888-018-1649-3 (PMC5907180; doi:10.1186/s12888-018-1649-3)
Supplement: Supplementary file 5 — Table S1. Life event items and corresponding abbreviations of the SEL interview. a open questions. (PDF 398 kb) [file 12888_2018_1649_MOESM5_ESM.pdf]

**Table S1: Life event items and corresponding abbreviations of the SEL interview**

| Abbreviation | Questions |
|---|---|
| **I Family Origin** | |
| H1 | Did you at any time in your life grow up in a family other than with your natural parents? |
| H2 | Did you grow up in a children's home? |
| | **The following questions now refer to your family or people who were important to you. (parents/stepparents/carers or brothers and sisters/stepbrothers and stepsisters/other children at the home)** |
| H3 | Did you ever have severe arguments or was there considerable tension over long periods of time in your relationship to your parents/those that were dear to you? (e.g. living together, school, studies, apprenticeship, friends, weren't respected, weren't understood, ignored, felt controlled). |
| H4 | Are your parents divorced? |
| H5 | Were there ever any arguments between your parents or people dear to you for longer periods of time that caused you distress? (at least 3 months) |
| H6 | Have you moved out of your childhood home? |
| H7[a] | Was there any other important event related to your family of origin that you have not yet mentioned, but that is very important to you? Which one? |
| **II Social Contact/Spare Time** | |
| S8 | Has a long-term friendship of yours ever broken up? |
| S9 | Were you generally very happy with the relationships to your friends? |
| S10 | Was there ever a period of time in which you were unhappy because you had to cut back on your leisure activities? (music, sport, reading, arts and crafts, club activities, car, travelling) |
| S11[a] | Were there any other important changes in the relationships to your friends or social contacts that you have not yet mentioned, but that are very important to you? Which ones? |
| **III Marriage and Relationships** | |
| B12 | Have you ever been unhappily in love or was a wish for a partner you longed for not fulfilled? (heartache) |
| B13 | How many serious relationships have you had? |
| | ***If you have never had a serious relationship, please continue to question 19!*** |
| B14 | When did you get married? |
| B15 | Have you ever been happy in your relationship for a longer period of time? (relationship was perceived as happy, harmonious and/or supportive for at least 3 months) |
| B16 | Have you ever had a serious crisis in a relationship? (Have you ever had a serious relationship crisis in your relationship as a couple?) |
| B17 | Have you ever been separated or divorced? (you or your partner stopped/ended the relationship after at least three months?) |
| B18 | Was a partner ever unfaithful to you in a relationship? |
| B19[a] | Was there any other important event related to your marriage or relationship that you have not yet mentioned, but that is very important to you? |
| **IV Pregnancy/Children** | |
| K20 | Have you ever suffered from not being able to fulfil your desire to have children? |
| | ***If you or your partner has never been pregnant or has no children, continue to question 29!*** |
| K21 | Have you or your partner ever given birth to one or more healthy children? |
| K22 | Have you or your partner ever given birth to a physically or mentally disabled child? |
| K23 | Have you or your partner ever had a miscarriage or given birth to a stillborn child? |
| K24 | Have you or your partner ever had an abortion? |

| | |
|---|---|
| K25 | Has your child or have your children ever moved out?<br>(e.g. due to marriage, arguments or for job reasons) |
| K26 | Did you ever have to be separated from your child against your will?<br>(e.g. due to illness, divorce, official order) |
| K27 | Did you ever have problems or arguments with your child/children (e.g. relationship, school) that strained you severely (at least 3 months)? |
| K28 | Did your child/children make you happy or very happy for most of the time (at least 3 months)? |
| K29[a] | Was there any other event related to your children that you have not yet mentioned, but that is important to you? Which one? |

**V School/Training and Further Education**

| | |
|---|---|
| L30 | Did you ever have to repeat a year or an important examination at school/during your traineeship or apprenticeship/during studies? |
| L31 | Have you ever completed a traineeship/apprenticeship or passed final examinations?<br>(traineeship/apprenticeship, secondary school leaving certificate, exam) |
| L32 | Have you ever quit a traineeship/apprenticeship or failed final examinations? (apprenticeship exam, intermediate school leaving certificate, exam) |
| L33 | Did you ever feel overburdened at school/during your apprenticeship/traineeship or whilst studying? (for a longer period of time, at least 3 months) |
| L34 | Did you ever have serious stress or confrontations with your fellow students or teachers/lecturers at your place of study or college? |
| L35 | Did you enjoy your training (school, apprenticeship/traineeship, studies) for most of the time or did it make you happy for at least 3 months? |
| L36 | Were you ever unable to start your desired course of training? |
| L37[a] | Was there any other event related to your training that you have not yet mentioned, but that is important to you? Which one? |

**VI Career/Household**
***If you have never had a job, please go to question 41!***

| | |
|---|---|
| A38 | Have you ever lost your job or been fired? |
| A39 | Have you ever been promoted or had significant professional success? |
| A40 | Have you ever had a serious failure in your professional career or been demoted at work? |
| A41 | Were your work tasks ever drastically changed? Or did you have to get used to a completely different task? (*If the change had to do with Reunification, please make note of it in the Reunification section*) |
| A42 | Have you ever been unemployed or unable to work for at least 3 months? |
| A43 | Did you ever have to do military/civilian service? |
| A44 | Have you ever had seriously strenuous relationships or confrontations with colleagues or superiors at work for a longer period of time (at least 3 months?) |
| A45 | Have you ever been forced into early retirement or a disability pension? (please note: semi-retirement, early retirement as having left paid employment prior to normal pension age) |
| A46 | Have you ever felt overburdened or severely strained due to your professional and or household tasks/duties for a longer period of time (at least 3 months)? |
| A47 | Have you enjoyed your professional or household tasks and have they made you very happy for most of the time? |
| A48[a] | Was there any other event related to your career that you have not yet mentioned, but that is very important to you? Which one? |

**VII Finances**

| | |
|---|---|
| F49 | Have you ever had considerable financial problems for a longer period of time (at least 3 months)? (e.g. not enough pocket money, not enough income, repayment of debt)? |
| F50 | Were you generally very happy with your financial situation for most of the time (at least 3 months)? |
| F51[a] | Was there any other event related to your financial situation that you have not yet mentioned, but that is very important to you? Which one? |
| | **VIII Living Situation** |
| W52 | Have you ever moved, built a house or done a large renovation to a house? |
| W53 | Did you ever find your living situation to be very strenuous for a longer period of time (at least 3 months)? |
| W54[a] | Was there any other event related to your living situation that you have not yet mentioned, but that is very important to you? Which one? |
| **VIII Court/Violations** | |
| J55 | Have you ever been sentenced to a prison sentence? |
| J56[a] | Was there any other event related to court or the police (legal proceedings, arrest, accusation, sentence) that you have not yet mentioned, but that is very important to you? Which one? |
| **IX Reunification**<br>***Find out whether the test person comes from former West Germany (A) or former East Germany (N)!*** | |
| D57 | Did the Reunification change the status of your professional career in a positive or negative manner? *(indicate the type of change with a "+" for a positive and a "-" for a negative change)* |
| D58 | Did the Reunification cause conflicts or separations (due to conflicts) in your family or your group of friends?<br>*(please note: record events related to family here, events related to friends under question 59. If only the family was affected, jump question 59).* |
| D59 | Did the reunification cause conflicts or separations in your group of friends? |
| D60 | Looking back, how would you rate the effects that the Reunification had on your life? (positive or negative) Have you gained more freedom (from a political point of view)? |
| D61[a] | Was there any other event related to Reunification that you have not yet mentioned, but that is very important to you? Which one? |
| **X Illness** | |
| G62 | Did you ever have to be admitted to a hospital or old peoples' home due to a serious bodily illness or an accident? |
| G63 | Have you ever been admitted to hospital for treatment of a mental illness? |
| G64 | Did anybody who is close to you (child, partner, parent, sibling) or a close friend ever have to be admitted to a hospital or old peoples' home due to a serious bodily illness or an accident? |
| G65 | Were you ever subject to considerably strenuous periods of time due to looking after or caring for (illness, disability, dependency) a close relation (parent, sibling) or a close friend? |
| G66[a] | Was there any other event related to your health that you have not yet mentioned, but that is very important to you? Which one? |
| **XI Deaths** | |
| T67 | Has one of your children ever died? |
| T68 | Has a partner of yours ever died? |

| | |
|---|---|
| T69 | Has a very important friend or relative, to whom you had a particularly close relationship and frequent contact, ever died? |
| T70 | Did a pet that you were very fond of ever die? |
| T71 | Have you ever tried to put an end to your life or commit suicide? |
| T72 | Did someone you loved or who was very close to you ever try to put an end to his/her life or commit suicide? |
| T73 | Did someone you loved ever commit suicide? |
| **XIII Accidents** | |
| **-> Show the list N1 of the PTBS section!!! Please note the numeration of the questions when entering the data!!!** | |
| U78 | Have you ever had a serious accident or suffered terrible experiences or a catastrophe as listed on the list N1?<br>- Been to war<br>- Threatened physically, attacked, injured, tortured<br>- Victim of rape<br>- Sexual abuse as a child<br>- Victim of a natural catastrophe<br>- Severe accident<br>- Held prisoner, held hostage, been kidnapped<br>- Forcedly displaced/forced to flee after WWII (question 81)<br>- Witness of one of these incidents |
| **->if none of the above occasions, finish the SEL and jump the PTBS section of the CIDI!** | |
| **XII Physical and Sexual Abuse** | |
| O74 | a) Were you ever physically abused or threatened by strangers (assault, attack)?<br>b) Were you ever sexually abused or threatened by strangers (assault, attack)? |
| O75 | a) Were you ever physically abused or threatened by somebody who was close to you?<br>b) Were you ever sexually abused or threatened by somebody who was close to you? |
| O76 | a) Have you ever witnessed physical abuse or the threatening of strangers?<br>b) Have you ever witnessed sexual abuse or the threatening of strangers? |
| O77 | a) Have you ever witnessed physical abuse or the threatening of persons who are close to you or you know well?<br>b) Have you ever witnessed sexual abuse or the threatening of persons who are close to you or you know well? |
| U79 | Have you ever witnessed a severe accident? |
| **XIV War** | |
| R80 | Have you ever been a victim of a military conflict or warfare? (e.g. affected by acts of war) |
| R81 | At the end of World War II, were you forcedly displaced from the former German eastern territories (e.g. Poland, Czechoslovakia, Yugoslavia after 1945)?<br>(forced displacement from the region of origin due to persecution, discrimination) |

2 [a] open questions
