# Supplementary figures and images for: Assessment of subjective emotional valence and long-lasting impact of life events: development and psychometrics of the Stralsund Life Event List (SEL)
Source: BMC Psychiatry. 2018 Apr 18;18:105. doi: 10.1186/s12888-018-1649-3 (PMC5907180; doi:10.1186/s12888-018-1649-3)

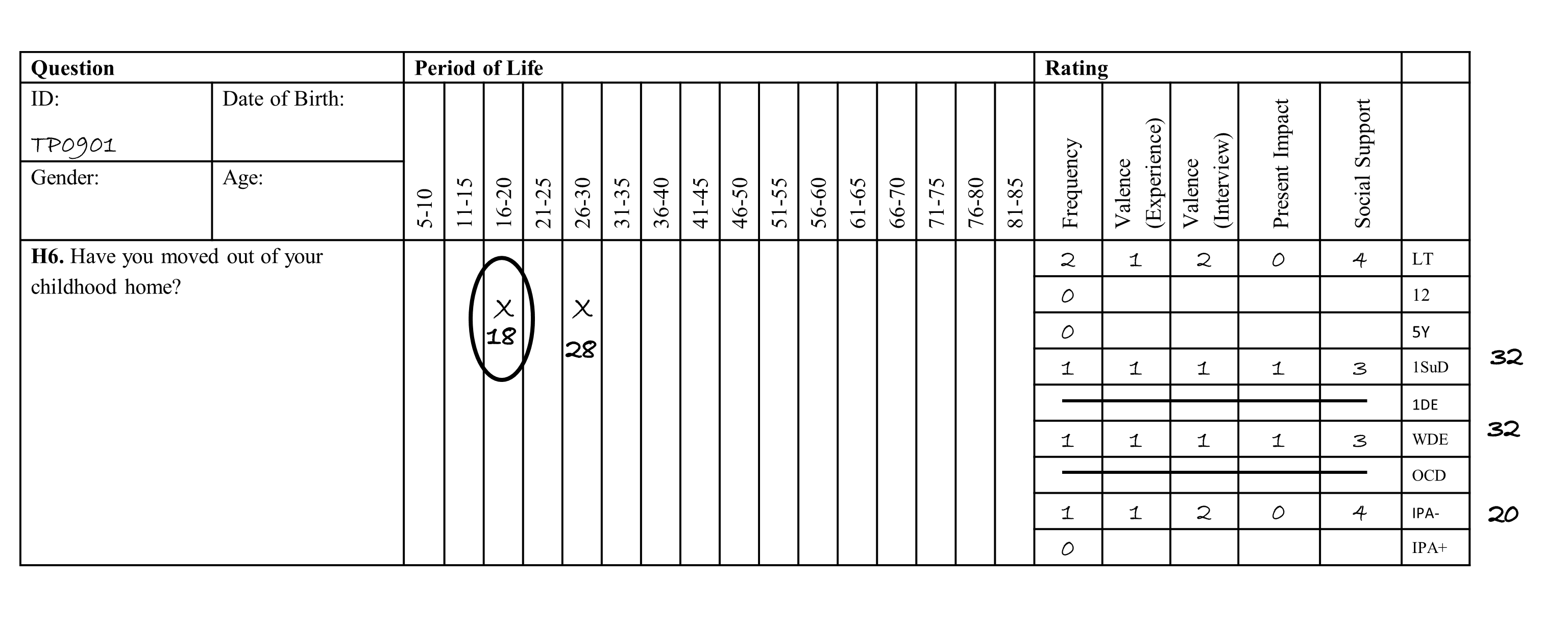

Supplement: Supplementary file 6 — Figure S1. Coding Example. A fictive person moved out of its childhood home twice (18 and 28 years). The most important experience was at the age of 18. The person had one subclinical depressive episode at the age of 32 and an initial panic attack at the age of 20. Hence, life event ratings concern the age of 18 for the whole lifespan and the 5-year period before the initial panic attack. For the 5-year period before the subclinical depressive episode, life event ratings concern the age of 28. LT = Lifetime; 12 = 12 months prior to the interview; 5Y = 5 years prior to the interview; 1SuD = 5 years prior to the first subclinical depressive episode; 1DE = 5 years prior to a clinical depressive episode; WDE = 5 years prior to the worst depressive episode; OCD = 5 years prior to an obsessive-compulsive disorder; IPA- = 5 years prior to an initial panic attack; IPA+ = 5 years after an initial panic attack. (TIF 249 kb) [file 12888_2018_1649_MOESM6_ESM.tif]
